# Activity-Cloud Organization of Shine–Dalgarno Sequences to Guide Translation Engineering in *Escherichia coli*

**DOI:** 10.1021/acsomega.5c09784

**Published:** 2026-02-01

**Authors:** Pavel Zach, Yadira Boada, Jesús Pico, Alejandro Vignoni

**Affiliations:** Synthetic Biology and Biosystems Control Lab, Instituto de Automática e Informática Industrial, Universitat Politècnica de València, València 46022, Spain

## Abstract

Modifying the six-nucleotide Shine–Dalgarno (SD) core motif inside the ribosome binding site (RBS) constitutes a straightforward approach for tuning bacterial translation. However, existing methods for adjusting the effective translation rate (ETR) lack predictability. Even single-nucleotide substitutions can induce substantial alterations in translation efficiency. Moreover, this unpredictability is exacerbated by variations in the leader sequence, spacer region, or coding context. By focusing on the SD core as a key, experimentally tunable determinant of translation initiation in *Escherichia coli*, we introduce a coarse-grained framework that organizes SD core variants into activity clouds with consistent expression levels. This representation converts a dense sequence-to-phenotype map into an interpretable design space for coarse-grained tuning of expression. In contrast to thermodynamic tools such as the RBS Calculator, which estimate initiation from biophysical parameters, our approach is data-driven and emphasizes (i) interpretable rules over nucleotide positions, (ii) a bidirectional workflow (core → expected ETR range; target ETR → candidate cores), and (iii) simple paths between clouds that suggest minimal sequence edits. Designed with the needs of research teams in mind, our workflow prioritizes fixing the SD core first (i.e., selecting an appropriate activity cloud) to substantially narrow the spread of observed ETRs across constructs. This dampens variability introduced by flanking DNA/RNA context (leader, spacer, local secondary structure), so that subsequent fine-tuning is simpler, cheaper, and more predictable. We validate cloud stability and predictive utility using an independent high-throughput data set. Our approach provides a solid foundation for fast, interpretable coarse control of expression, while fine-grained tuning can then be achieved through flanking-region edits that account for spacing and local structure. Finally, we provide an open web interface and repository, allowing researchers to explore the hierarchy, inspect positional influences, and export candidate cores. Together, these contributions advance the Bonde et al. data set from a static lookup into a portable, actionable map for SD core guided tuning of translation in *E. coli*, and outline a path to extend the idea to a fully functional framework, with possible applications also to other bacteria.

## Graphical abstract

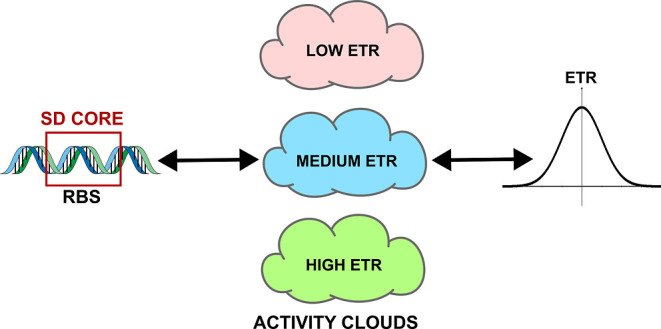

## Introduction

Initiation of bacterial translation emerges from the interplay of several determinants, including hybridization between the mRNA Shine–Dalgarno motif and the 16S rRNA anti-SD (ASD), the spacing between the SD and the start codon, local mRNA secondary structure, and early coding-sequence context.^1^ While the six-nucleotide SD core is often a strong, tunable contributor to expression in *Escherichia coli*,^2,3^ it is not an independent driver of initiation in isolation. Mechanistically, the constant ASD core in 16S rRNA (e.g., CCUCC, nt 1535–1539) pairs with variable mRNA SD sequences,^4^ and prior work has highlighted AGGA-like motifs with context-dependent spacing relative to AUG.^5^ Moreover, SD usage varies across bacteria,^6^ with some lineages lacking recognizable SDs.^7^ Even within *E. coli*, the correlation between SD “strength” and translation efficiency can be modest in natural genes.^8^ In this study, we therefore focus explicitly on the SD core projection of the initiation landscape in *E. coli* for the synthetic biology applications, treating other determinants as known covariates that can be layered on for fine control.

Thermodynamic models such as the RBS Calculator or UTR Designer estimate initiation rates from biophysical parameters (e.g., Δ*G* of rRNA–mRNA pairing, standby sites, and local structure) and are widely used in design.^9,10^ Orthogonally, large-scale empirical maps have shown that single-nucleotide edits within the SD core can tune expression in a fixed 5′-UTR (untranslated region) context.^3^ However, such predictability often degrades when leaders, spacers, or coding sequences change.^10–12^ Prior guidance has thus emphasized choosing cores expected to yield similar expression while minimizing perturbations to mRNA folding,^3^ or fixing the SD core and optimizing the SD-flanking sequences.^10^

Building on these insights, we introduce a structure-informed, data-driven approach that organizes all 4096 six-mer SD cores into a pruned hierarchical tree and partitions them into “activity clouds,” i.e., sets of cores with statistically almost indistinguishable effective translation rate (ETRs) in *E. coli*. This representation converts a dense sequence-phenotype map into an interpretable, coarse-grained design space. Practically, it enables a bidirectional workflow: (i) forward mapping from a chosen core to an expected ETR range, and (ii) inverse mapping from a target ETR to candidate cores within an appropriate cloud. Within a chosen cloud, fine-tuning can then proceed via flanking-region edits (leader, spacing, local structure, and first codons) using iterative DBTL (Design-Build-Test-Learn) cycles or thermodynamic guidance; such edits typically adjust ETR within the cloud’s range, while core changes move between clouds. In comparison to only fixing the SD core (e.g., always using the canonical SD sequence) and using DBTL cycles to tune the ETR, the prior selection of the cloud and its SD cores should reduce the number of DBTL cycles by narrowing the possible ETR range.

We position this proposed method as complementary to mechanistic tools. Whereas thermodynamic models aim to predict absolute initiation by explicitly modeling Δ*G* and spacing, our approach provides (a) portable, interpretable coarse control overexpression via SD core choices, (b) positional rules that summarize nucleotide-by-position influence, and (c) simple sequence paths linking clouds that suggest minimal core edits for stepwise tuning. We validate cloud stability and predictive utility using an independent high-throughput data set, and we benchmark against thermodynamic estimates to demonstrate complementarity rather than redundancy. Finally, to support community use and reproducibility, we provide an open web interface and a repository, enabling researchers to explore the hierarchy, inspect positional influences, and export candidate cores.

In summary, our contribution is to elevate the Bonde et al.^3^ data set from a static lookup table to a portable, actionable map for SD core-guided, coarse-grained tuning of translation in *E. coli* for synthetic biology engineering applications, while explicitly acknowledging and providing a pathway to integrate the additional biophysical determinants that govern initiation.

Crucially, our approach provides a bidirectional map between SD core sequence space and translation output (see Figure 1), while providing additional evolutionary insights that could be used for further exploration in this area.

**Figure 1:**
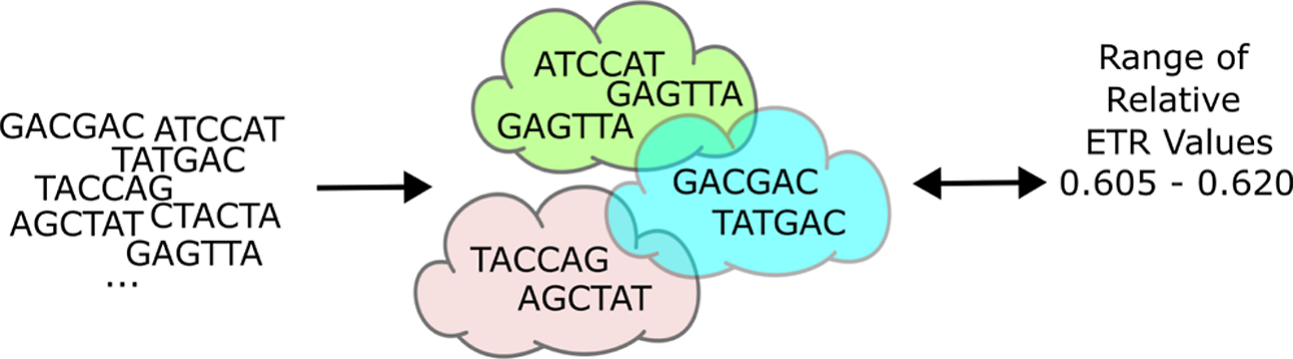
Schematic of the described approach to tune the ETR value of the RBS. Given an input SD core, the model predicts the corresponding range of ETR values by identifying the activity cloud to which the SD core belongs. Conversely, for any target ETR value, the framework identifies and selects an appropriate activity cloud(s) with SD cores that the user can choose from.

Moreover, analysis of the final pruned hierarchical tree illuminates why single-nucleotide differences can produce dramatic shifts in ETR, whereas cores with low sequence similarity may display convergent translation efficiencies. This hierarchical organization thus aims to restore robust, context-insensitive control over translation in *E. coli*, setting the stage for precise multielement expression tuning.

Importantly, the Activity-Cloud framework is not intended to eliminate or replace the well-known context dependence of bacterial translation initiation. Instead, it provides a structured, coarse-grained representation of SD core sequence space that captures consistent expression bands across a fixed genetic context. In this sense, activity clouds should be interpreted as iso-functional regions that constrain expected translation efficiency to a narrow range, rather than as predictors of absolute expression across arbitrary sequence environments. Contextual effects arising from mRNA secondary structure, spacer length, or coding-sequence interactions are deliberately deferred to downstream fine-tuning steps within a DBTL workflow.

## Results and Discussion

### Tree Construction and Pruning

We began by representing all 4096 possible SD core sequences using an abstract notation in which each underscore (“_”) denotes an unspecified (wildcard) nucleotide. At Level 0, the root node is written as “______,” (corresponding to a fully undefined six-nucleotide SD core), and at each subsequent level, each underscore is sequentially replaced by one of the four canonical nucleotides (A, T, G, C—see the Methods section for a detailed description and Figure 2 for a schematic view of the tree). Iterating this process through seven levels yields a complete hierarchy whose leaves correspond to fully specified SD cores.

**Figure 2:**
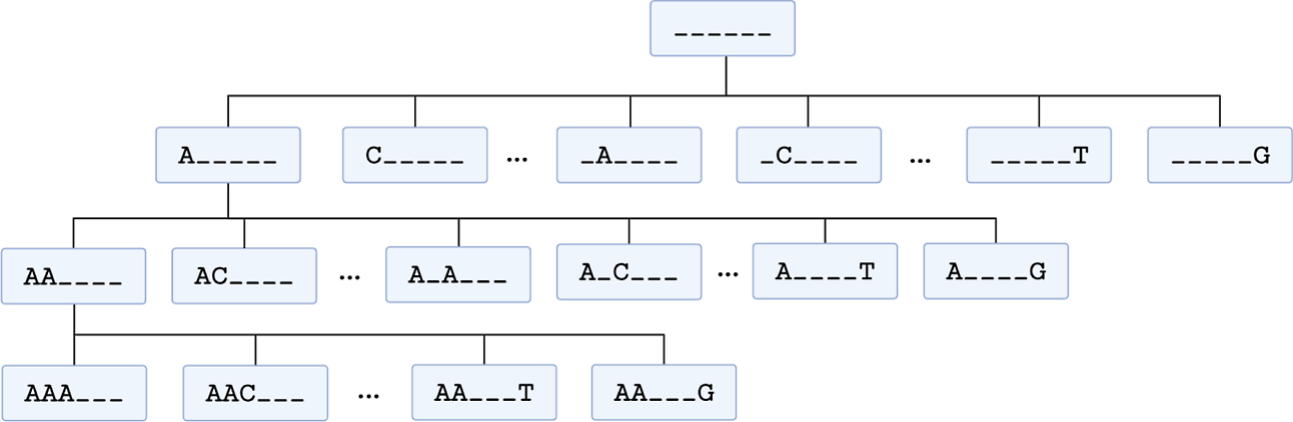
Schematic of the hierarchical tree over six-mer SD sequences. For clarity, only levels 0–3 are shown, with expansion illustrated along the leftmost branch and a few representative nodes at each level.

Because any given sequence can be reached via multiple paths (e.g., AAAAAA arises from _AAAAA, A_AAAA, AAAAA_etc.), we imposed a pruning procedure to ensure each terminal sequence appears exactly once, following the path of minimal variability. First, we annotated every leaf with its measured ETR from Bonde et al.^3^ and propagated both the mean ETR and the coefficient of variation (CV) upward by averaging over child nodes. Then, using a depth-first traversal, we iteratively removed intermediate nodes contributing disproportionately to high CV while preserving any branch whose leaf sequences were already uniquely represented, until no further reduction was possible. The result is a single, nonredundant path from root to each SD core that minimizes CV across the hierarchy, as shown in Table 1.

**Table 1:** First Ten Branches of the Pruned Tree, Showing the Path from the Level 0 up to the Specific, Unique SD Core Sequence at Level 6^a^

level 0	level 1	level 2	level 3	level 4	level 5	level 6	ETR
______	A_____	AA____	AAT___	AATT__	AATTT_	AATTTA	0.1190
______	A_____	AA____	AAT___	AATT__	AATT_T	AATTCT	0.1175
______	A_____	AA____	AAT___	AAT_C_	AATTC_	AATTCA	0.1068
______	A_____	AA____	AA_T__	AA_TT_	AA_TTA	AACTTA	0.1186
______	A_____	AA____	AA__C_	AA__CC	AAA_CC	AAATCC	0.1145
______	A_____	AA____	AA__T_	AAT_T_	AATAT_	AATATG	0.1336
______	A_____	AA____	AA__T_	AAT_T_	AAT_TT	AATATT	0.1334
______	A_____	AA____	AA__T_	AA_CT_	AA_CTC	AAACTC	0.1208
______	A_____	AA____	AA__T_	AA__TA	AAC_TA	AACATA	0.1273
______	A_____	AA____	AA__T_	AA__TC	AAA_TC	AAAATC	0.1204
______	A_____	AA____	AA____C	AA_C_C	AA_CGC	AACCGC	0.1177

a Last column represents the ETR of this particular SD core from the EMOPEC dataset.^3^

At this stage, we deliberately avoided conventional clustering or supervised learning approaches, as the modest size of the sequence space and the imperative to preserve sequence-to-structure relationships rendered them unnecessary. Instead, our pruned tree offers a compact feature set for downstream machine learning. By filtering out sequence contexts with high variability, the tree highlights consistent SD core motifs and reveals that small branch distances correlate with minor ETR differences, whereas even single-nucleotide variants may diverge sharply if they lie on distant branches. This structure-informed approach, therefore, not only identifies robust sequence clusters but also illuminates the interplay between SD core identity and translation efficiency.

A decision tree regressor was trained to visualize and interpret the rules extracted from the pruned hierarchical tree. The resulting rules elucidate the governing principles underlying the core ETR by specifying which nucleotide identity at each hierarchical level exerts the most significant influence.

### Activity Cloud Partitioning

After pruning, we compiled a data frame containing every node from level 1 through level 6, each annotated with its six-character SD pattern (including the underscore wildcards), and its measured ETR. To capture sequence variation, each position was one-hot encoded using five possible symbols (A, C, G, T, and “_”), yielding a 30-column binary matrix. The raw ETR values were encoded in the form of additional columns, ensuring that sequence features and translational efficiency were weighted comparably during clustering (see the Methods section for more details).

We next applied the Density-Based Spatial Clustering of Applications with Noise (DBSCAN) algorithm to this augmented data set, using a Euclidean distance metric and empirically optimized neighborhood radius ϵ and minimum–samples parameters. DBSCAN’s ability to identify clusters of arbitrary shape and to designate sparse points as noise makes it particularly well-suited to our heterogeneous data. The resulting “activity clouds” group SD cores that are proximal both in sequence space and in normalized ETR. An excerpt from the resulting data table can be seen in Table 2.

**Table 2:** First Five Rows from the Activity Cloud ETR Statistics^a^

activity cloud #	ETR mean	ETR STD	core sequences
0	0.1296	0.0174	AATTTA, AATTCT, AATTCA, AACTTA, AAATCC, AATATG, AATATT, AAACTC, AACATA, AAAATC, AACCGC, ATGCAA, ATACCA, ATCCGT, ATACAT, ATCGAT, ATGCGC
1	0.2290	N/A	ATCCGC
2	0.1234	0.0146	ACCTAC, ACCTCC, ACATCC, ACATCA, ACATAC, AAATCA, ATATTA, AAATTA, AAATCG, ATCTAA, ATTTGA, ATTTAA, ATGTCT, ATCTCT, ACCTAT, ATATAT, ATCTTT, AACTAA, AAATAC, AACCAA, AATCAA, AAACAA, AATCAG, ATTCAG, AAACAT, AACCAG, ATATAA, ACATAA, ACGCAC, ACTCAC, ACGGAC, ATAAAC, AACCAC, ACAAAC, AAAAAC, ATATCA, ACTCTA, ACACTG, ACAATG, ATACTC, ATACTT, AACATC, ACCATA, ACGATC, ATGATC, AAAATA, ATTTTA, ATCTTA, AACACA, AACAAA, AAACCA, ACTAAA, ACCGCA, AAAATT, AAACCT, AACGTT, ACATAT, ACAAGT, ATACCT, AATACT, ACTAAT, ATTCAT, AATCAT, ACTGCT, AAATCT, ACCTGT, AATTGT
3	0.0528	0.0084	CGTTCC, CGTCCC, CGTCAC
4	0.1048	0.0133	CCGAAA, CCGAAT, CCGTGA, CTGCCT, CCGGAC, CAAGTC, CCGGCC, CCGGTC, CCAGCC, CCTGTC, CCCGAC, CACGAA, CGGGAC, CTTGTA
5	0.0800	0.0205	CATAGT, CCACGT, CAACGC, CCGCGA, CTGCGC, CTCTGC, CTTTGC, CAACGA, CCACGA, CATTGT, CCTTGT, CATCTA, CATATG, CAAATC, CACCTG, CGCCTA, CGCATG, CGTCTT, CGTCTC, CCGTTG, CTGTTT, CCGGTG, CAGCTG, CATGTC, CTTGTT, CTTGTG, CACGTG, CTCTTG, CGCTTG, CATTTA

a Each row represents a single activity cloud, with the mean ETR values of Its SD core sequences, together with the standard deviation.

Mechanistically, we hypothesize that a cloud represents an iso-energetic basin in the SD-ASD free-energy landscape. Because the ribosome “reads” net binding energy rather than primary sequence,^13^ variants scattered in sequence space can collapse onto the same translational output.

Consistent with Zhang et al.,^2^ cores within the same cloud exhibit similar translation rates across diverse 5′-UTR contexts when the mRNA secondary structure and its energy remain consistent. Therefore, the core sequence can be chosen from the activity cloud so that the free energy of the mRNA secondary structure remains unchanged, allowing for a predictable, coarse-grained ETR. Subsequently, the flanking regions can be refined via iterative DBTL cycles to achieve precise expression tuning without compromising overall efficiency. Metaphorically, it is like choosing a cloud by its centroid (the mean ETR) and then moving inside that cloud via targeted flanking-region edits, as shown in Figure 3.

**Figure 3:**
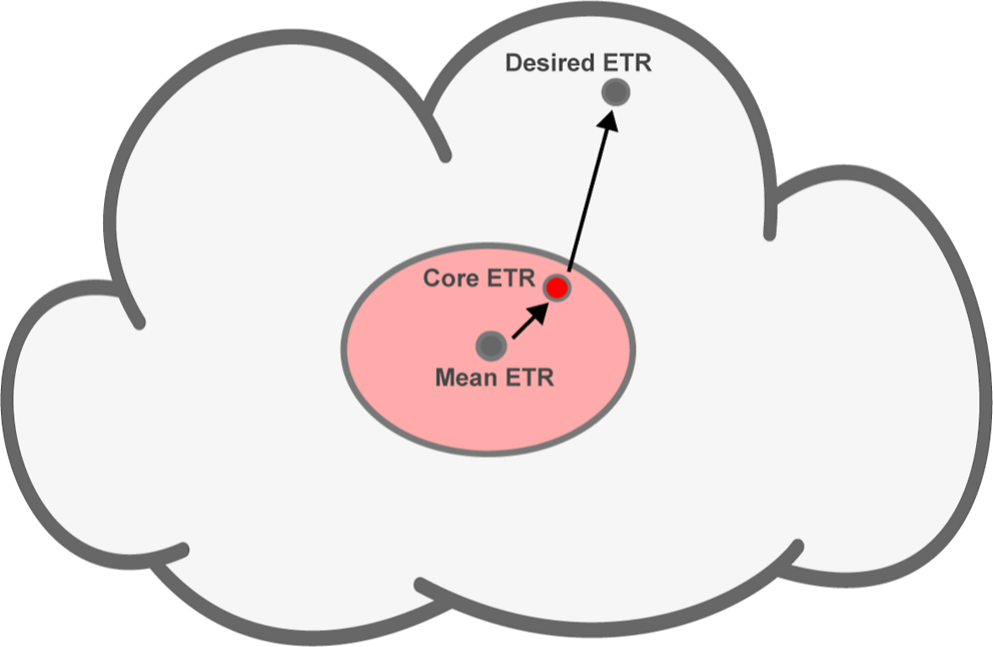
An activity cloud is characterized by its mean ETR and its constituent SD core sequences. Once one or more activity clouds matching the target ETR have been identified, an individual SD core is selected from the chosen cloud—preferably one that yields the same local mRNA secondary structure, i.e., the same Δ*G* in the translation-initiation region. Finally, flanking-region edits can be applied to fine-tune the ETR to the desired value using DBTL cycles.

### Interactive Web Interface for Exploration

To facilitate reproducibility and enable intuitive exploration of the sequence space, we developed an interactive web application that visualizes the hierarchical tree, pruned branches, and resulting activity clouds. The interface allows users to navigate from any partially specified SD core motif to its descendant sequences, inspect the corresponding expression values, and view cluster memberships assigned by DBSCAN.

Each activity cloud is rendered as an interactive plot where users can highlight individual sequences and display their associated mean ETR, CV, and relative cluster position.

The web interface was implemented in Flask and uses precomputed CSVs from the pruning and clustering workflows (see the Methods section). It is available at http://sb2cl-vm1.ai2.upv.es/.

This interactive tool enables researchers to dynamically explore the structure–function relationships within the SD core, extending beyond the static visualizations presented in this manuscript. An example of the web interface is shown in Figure 4.

**Figure 4:**
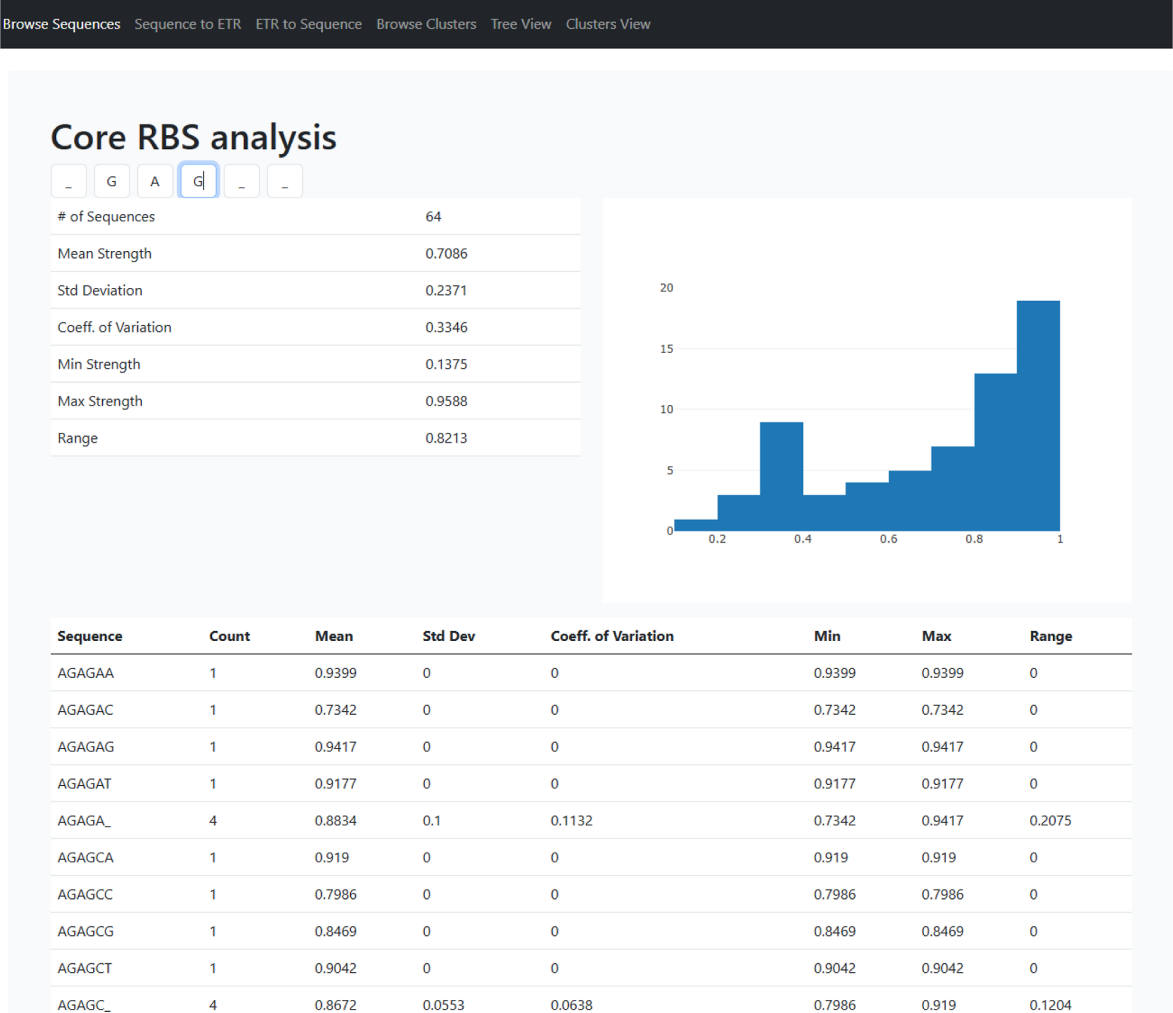
Screenshot of the web application, illustrating the visualization of SD cores and their ETRs. By substituting the wildcard underscore characters with specific nucleotides, the data set is automatically filtered, and the corresponding statistics are shown.

A detailed description of the interactive web application is provided in the Supporting Information (Supporting Information, Section S2). The online tool allows users to explore and apply the Activity-Cloud framework through several interactive modules that reproduce the analyses reported in this work. Users can browse all possible six-nucleotide Shine–Dalgarno (SD) cores and obtain summary statistics for user-defined nucleotide patterns (Section S2.1), predict expression levels from a given 5′-UTR sequence by identifying and mapping its SD core to the corresponding activity cloud (Supporting Information, Section S2.2), or inversely retrieve representative SD cores associated with a target expression rate (Supporting Information, Section S2.3). Additional views enable browsing of all cores and cloud statistics (Supporting Information, Section S2.4), interactive visualization of the pruned tree hierarchy (Supporting Information, Section S2.5), and a dynamic map summarizing mean expression and variability across activity clouds (Supporting Information, Section S2.6). Further implementation details and links to the complete data set, Jupyter notebooks, and source code used to reproduce all analyses are available in the Supporting Information (Supporting Information, Section S3, Data and Code Availability).

### Validation with μASPIre Data Set

To test cross-context robustness, we compared cloud-predicted ETRs against the μASPIre data set’s integral-flipping metric (IFP_0–480 min_).^11^ This metric represents the normalized area under the flipping-profile curve from 0 to 480 min after induction, used by the authors as a quantitative summary of RBS activity that correlates with cellular protein levels.^11^ While individual cores exhibit context-dependent shifts, their ETRs remain mostly confined around each cloud’s range, confirming reliable coarse-grained control (Figure 5).

**Figure 5:**
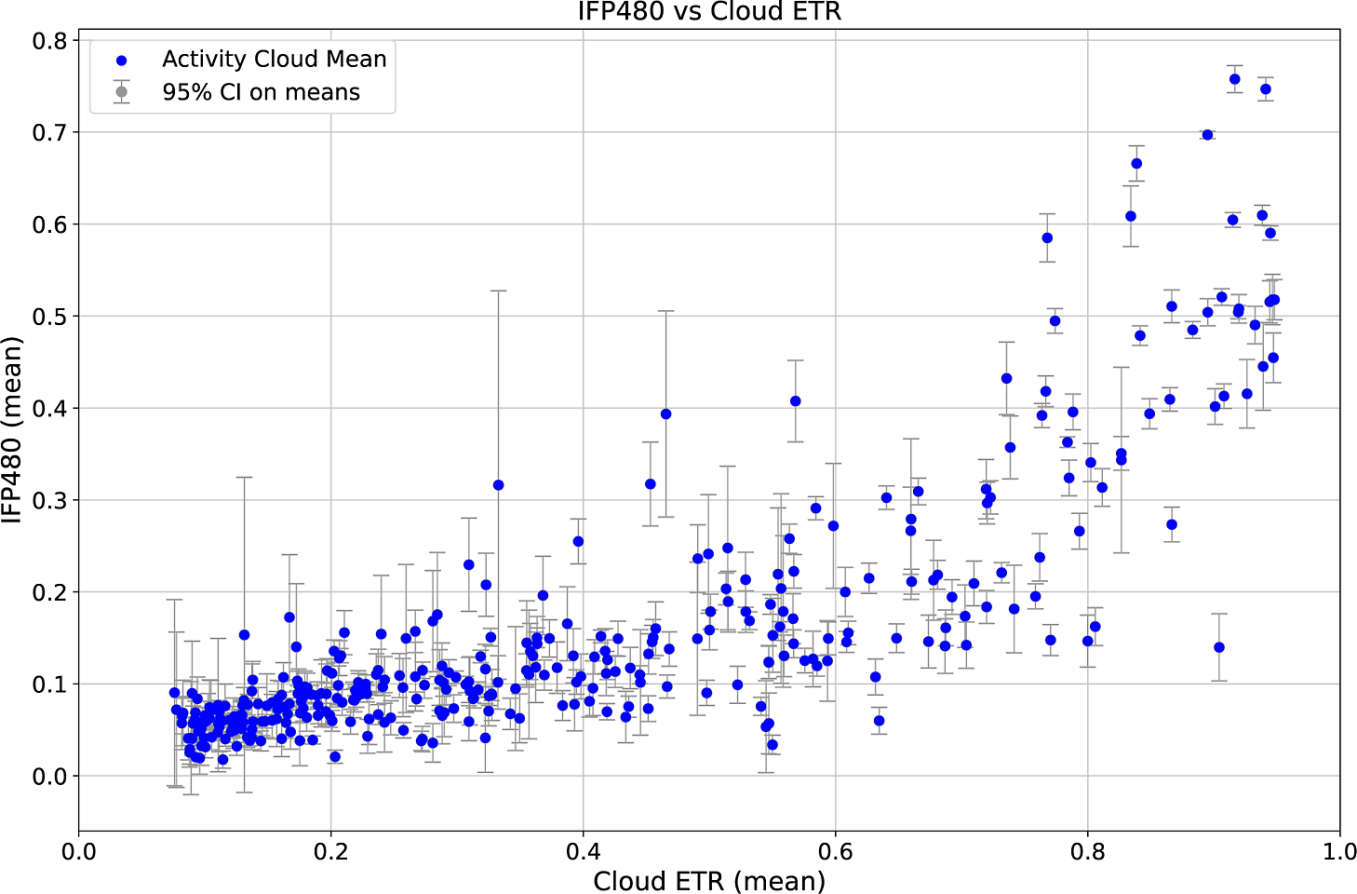
Scatter of mean IFP480 (*y*; normalized integrated fluorescence over 0–480 min) versus cloud-mean ETR (*x*) for all activity clouds. Each point represents one activity cloud. Vertical error bars show 95% confidence intervals of the cloud means. Cloud-level aggregation (sequence to cloud) reduces context noise and reveals a strong positive association between predicted ETR and experimental value (IFP480), with increasing dispersion at higher activities. Consistent with expectations for random SD-motif libraries, most clouds occupy the low-ETR regime, indicating that the majority of SD variants confer weak initiation.

We assessed the linear association between IFP_0–480 min_ and the ETR of each determined activity cloud by computing the Pearson correlation coefficient, yielding

$$r=0.81893,\;p=3.54\times 10^{-86}$$

The coefficient *r* = 0.81893 indicates a strong positive linear relationship, which means that higher IFP_0–480 min_ values correspond to higher values of the activity cloud ETR, although the fine-grained variability remains unexplained by clouds alone. Moreover, the extremely small *p*-value (*p* ≪ 0.001) provides overwhelming evidence against the null hypothesis of zero correlation. Thus, the observed correlation is highly statistically significant.

### Validation with the RBS Calculator

To further evaluate our approach, we compared our predictions with those from the RBS Calculator, a state-of-the-art thermodynamics-based tool for estimating translation initiation rates.^9^

As outlined in Implications and Limitations, we expected the best agreement for RBSs with weak local mRNA secondary structure, and a progressive deterioration of agreement for RBSs with strong secondary structure. As a starting point, we used the 5′-UTR from our experimentally characterized plasmid that constitutively expresses GFP. From this template, we generated all variants obtained by mutating the six nucleotides immediately upstream of a fixed 7 nt spacer. From this variant set, we selected sequences from the extremes of predicted mRNA folding stability (see the Methods section for details), covering the whole range of ETR values.

For each sequence, the SD core was identified as the 6-mer within the canonical search window that exhibited the strongest SD-ASD interaction (most favorable Δ*G*), with permissible SD-AUG spacer lengths of 5–8 nt. We then compared the cloud mean ETR associated with the identified core to the translation initiation rate predicted by the RBS Calculator (Figure 6).

**Figure 6:**
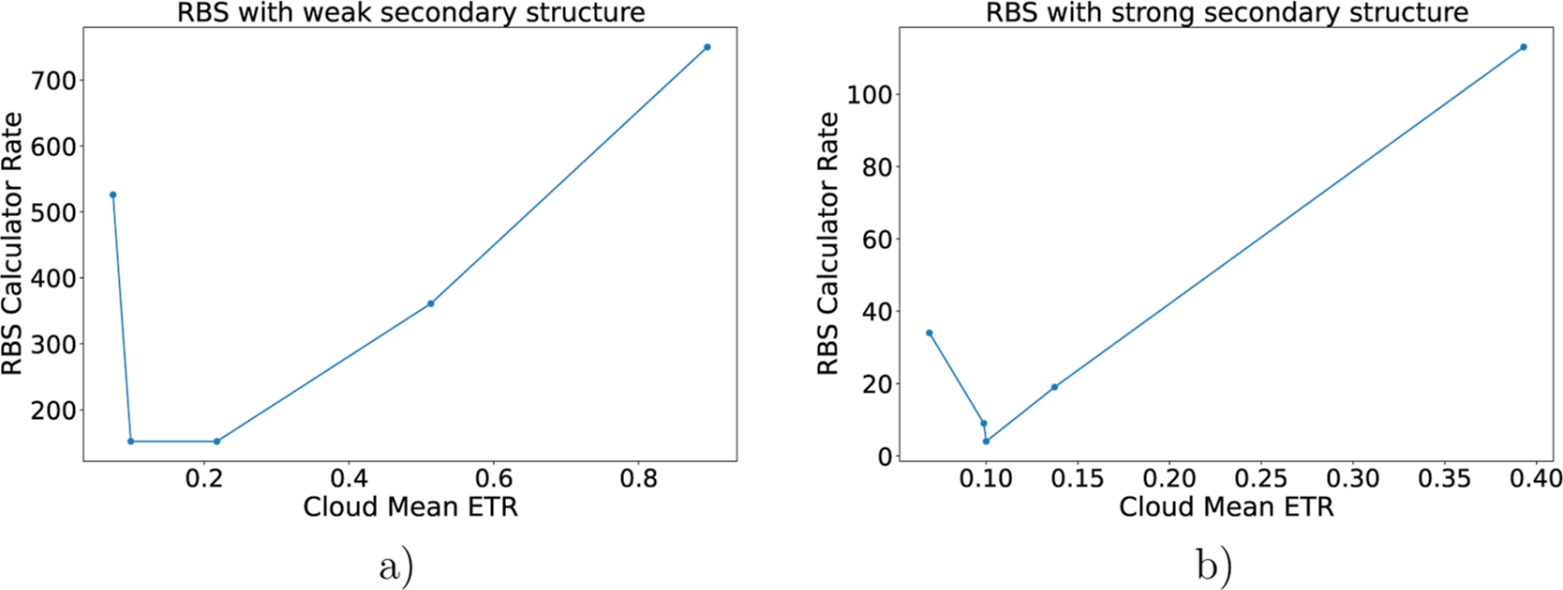
Comparison of mean cloud ETR with RBS Calculator across mRNA structural regimes. Both panels use the same GFP 5′-UTR template with all 6-mer substitutions immediately upstream of a fixed 7 nt spacer; see Methods for selection of weak/strong structure sets and SD core identification. (a) RBS with weak secondary mRNA structure: points show the activity cloud mean ETR vs the RBS calculator prediction. Agreement is generally good for higher ETRs. (b) RBS with strong secondary mRNA structure: the RBS calculator tends to predict higher initiation rates than the cloud-based estimates in the low-ETR regime, consistent with increased influence of non-core RBS features (secondary structure, precise spacing, early CDS context).

In general, the predicted strengths were in good agreement for mean cloud ETR values ≥0.15. For lower ETRs, the RBS Calculator tended to predict higher initiation rates than our coarse-grained approach. A plausible explanation is that, in these weak-core regimes, noncore RBS features (e.g., local secondary structure, exact SD-AUG spacing, and early coding-sequence context) exert proportionally larger influence than the SD core alone, leading to systematic upward deviations of thermodynamic predictions relative to our cloud-level estimates.

### Positional Nucleotide Influence from PLS Regression

To quantify how each nucleotide at each position within the six-base SD core contributes to the ETR, we fitted a partial least-squares (PLS) regression model using the one-hot encoded nucleotide identities (A, C, G, T, and “_” wildcard) as predictors, and the ETR as the response. After splitting the data set into an 80% training set and a 20% test set, we selected four PLS components, balancing model complexity and predictive accuracy. This yielded a coefficient for every nucleotide-position combination.

The PLS regression model was chosen since it can inherently handle multicollinearity among the one-hot encoded categorical variables, while also offering more stability when dealing with highly correlated predictors. While LASSO (Least Absolute Shrinkage and Selection Operator) is another regularization technique that can handle multicollinearity (by shrinking some coefficients to zero and performing variable selection) PLS provides components that can offer insights into the multivariate structure of the data and its relation to the response. Since our goal was to quantify the influence of each nucleotide at each position, PLS’s ability to create a set of orthogonal components and then attribute contributions through these components is highly advantageous for interpretation.

We then aggregated the original and absolute values of these coefficients across all components for each nucleotide at each of the six positions, yielding a “total influence” metric. As shown in Figure 7, guanine (G) at positions 3 and 4 exhibits the largest positive contributions to ETR, consistent with the known preference of the 16S rRNA ASD helix for G-rich motifs in the SD core.^2^ Adenine (A) and thymine (T) residues contribute moderately but more weakly, while cytosine (C) shows intermediate effects. The relative heights of the stacked bars confirm that the central positions of the six-nucleotide core exert the greatest overall influence on translation initiation. In contrast, the terminal positions play a smaller role. This dominance of the central dinucleotide explains why clouds tolerate peripheral mutations. Variations at positions 1 or 6 modulate the stacking energy only marginally, leaving the core G-rich interaction and, thus, the overall ETR largely intact.

**Figure 7:**
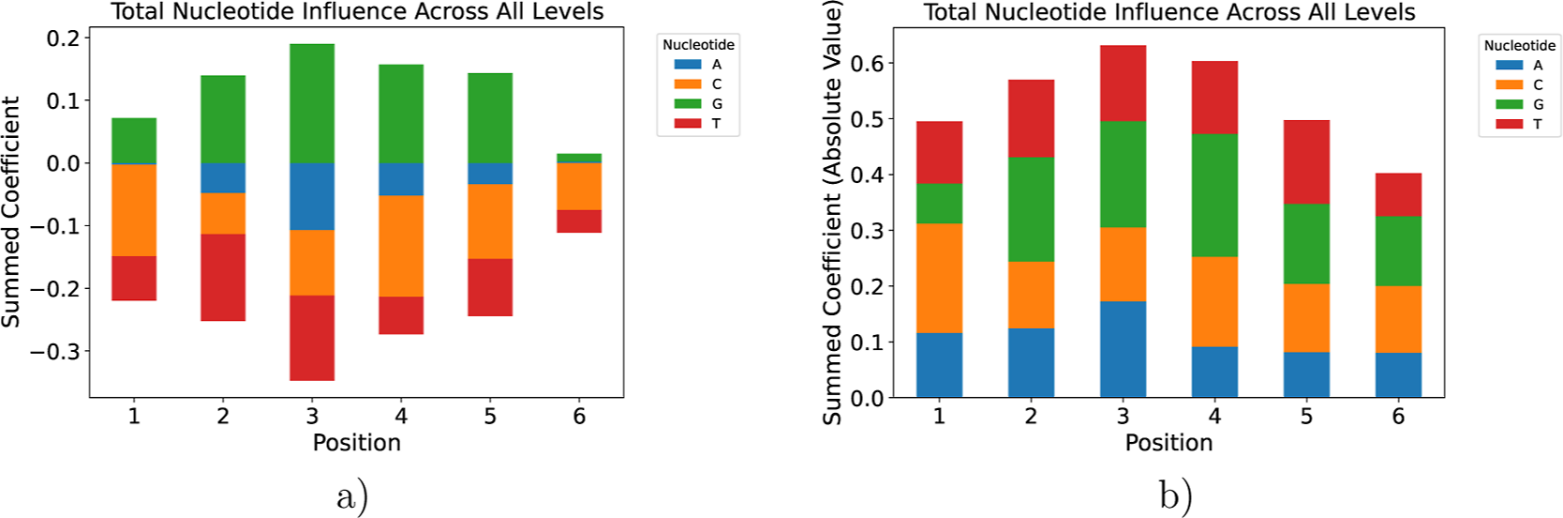
Influence of each nucleotide at each SD-core position. The higher the value for a particular nucleotide at a given position, the higher the influence. While guanine exhibits almost entirely a positive influence, adenine has a neutral impact at the beginning and at the end of the SD core. (a) Total influence of each nucleotide at each SD-core position, derived from the sum of PLS regression coefficients. (b) Total influence of each nucleotide at each SD-core position, derived from the sum of absolute PLS regression coefficients.

Note that the bars in this plot do not sum to 1 at each position, since these values are not proportions or probabilities, but rather the raw magnitudes of influence. Each bar height represents the sum of PLS regression coefficients for that nucleotide-position feature across all components. A larger magnitude indicates a stronger effect (positive or negative) on the predicted ETR, and the total stack height per position reflects the overall importance of that position for translational efficiency.

These quantitative insights derived from PLS analysis reinforce the structural principle that both nucleotide identity and positional context within the SD core jointly determine hybridization strength and, consequently, translational efficiency. Quantified contributions, together with the results of structural analysis, could provide a foundation for future work aimed at elucidating additional aspects of the structure–function relationship within the RBS core.

As shown in Figure 8a, these results are consistent with the analysis of the pruned tree that reveals the influence of G nucleotides in the SD core sequence. Guanine residues occur at the very beginning of more than 60% of all branches, underscoring the strong impact of this nucleotide.

**Figure 8:**
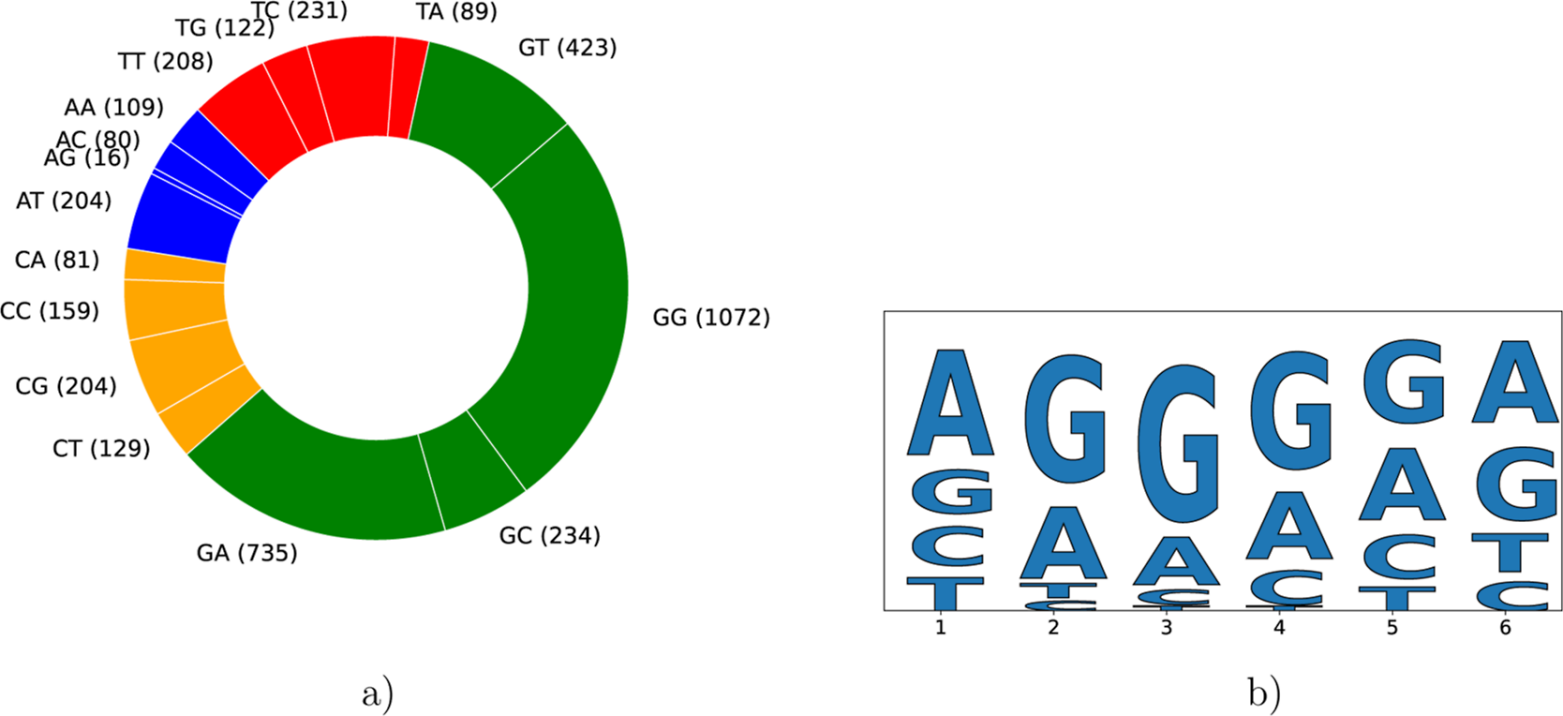
Analysis of the impact of different nucleotides on the final ETR level, revealing the main determinants. (a) Distribution of nucleotides in Level 1 and Level 2, showing that the major determinant of the ETR value is the presence of the Guanine nucleotide. (b) Distribution of the nucleotides in activity clouds with the mean ETR > 0.75. Presence of the Guanine nucleotides in the center of the SD sequence is a strong determinant of a high ETR value.

### Mutational Connectivity and Evolutionary Accessibility of Activity Clouds

We modeled the activity cloud design space as a single-nucleotide (Hamming-1) graph in which nodes are the SD core sequences and edges connect sequences differing by one base. Overlaying activity-cloud labels revealed that most clouds form connected neutral networks: within-cloud graphs typically contain a large giant component, indicating that many one-step mutations preserve coarse expression (see Figure 9).

**Figure 9:**
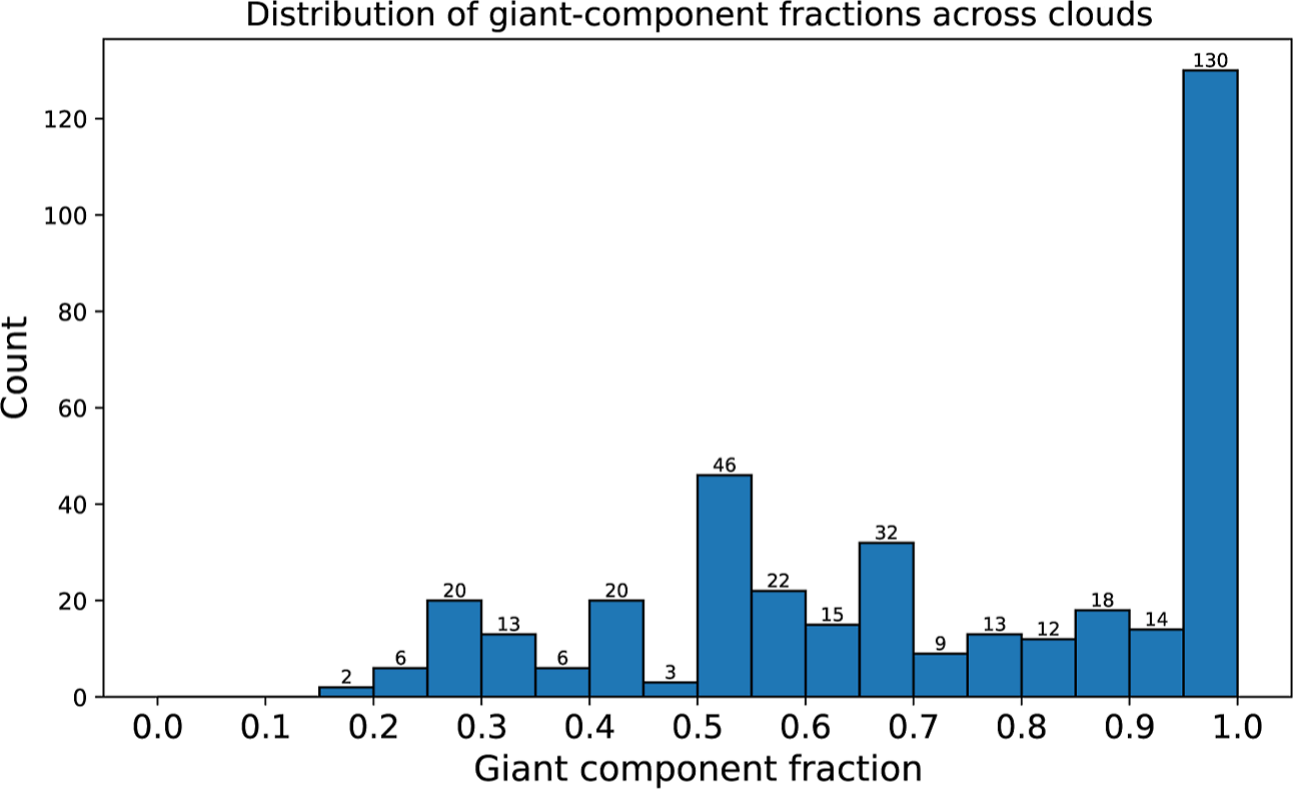
Histogram of giant-component fractions across activity clouds. A larger fraction indicates greater within-cloud mutational robustness—i.e., under single-nucleotide (Hamming-1) changes to the SD sequence, more cores remain connected within the same cloud (preserve the same coarse expression class). A high count at 1.0 means many clouds have an induced subgraph in which all (or nearly all) cores lie in one connected component, consistent with strong robustness to one-step SD mutations.

Collapsing the sequence graph to a cloud meta-graph, where nodes represent clouds and edges represent one-step “bridges” between clouds, showed extensive intercloud connectivity (see Supporting Information, Figure S1). We quantified the typical steepness of cloud boundaries using the mean absolute |ΔETR| across bridges (Supporting Information, Figure S2), which highlighted both near-neutral boundaries (transitions where the change of ETR is small) and sharp transitions (transitions that produce substantial ETR shifts).

To assess evolutionary accessibility, we computed a row–stochastic transition matrix *P*(*C*_*i*_ → *C*_*j*_) under a random single-base step (Supporting Information, Figure S3). Diagonal entries estimate *P*(stay) (robustness within a cloud), while off-diagonals indicate where mutations go when they leave. We also measured the fraction of beneficial moves (ΔETR > 0) for each ordered cloud pair. Finally, restricting to edges with nondecreasing ETR (ΔETR ≥ 0), we identified short, selectively accessible paths from low- to higher-ETR clouds, demonstrating practical, stepwise routes for tuning. Together, these results show that activity clouds are not isolated clusters but are connected by multiple one-mutation bridges, offering both robustness within clouds and evolvability via short, often beneficial exits.

### Implications and Limitations

We demonstrated that substitution of the SD core sequence alone does not afford precise, absolute control over translation initiation. Nevertheless, it can provide sufficient modulation for applications demanding only coarse adjustment within a constant or closely related 5′UTR context or serve as a method for efficiently decreasing the number of the DBTL cycles needed. The empirically derived relationships between the SD core sequence and ETR furnish useful guidelines for initial RBS design. Once an appropriate core SD sequence is selected from an activity cloud to achieve a target ETR, subsequent DBTL iterations focused on flanking regions outside the core can be employed to fine-tune translation output in the desired direction, while the ETR range imposed by the activity cloud limits the number of DBTL cycles needed. This is, by construction, activity clouds do not predict absolute translation rates across arbitrary genetic contexts; rather, they reduce the effective design space by grouping SD cores into robust expression bands, within which context-specific optimization can be subsequently performed.

While this study focuses on *E. coli*, the workflow is organism-agnostic and can be ported by retraining cloud assignments with organism-specific data and updating spacing/structure priors (e.g., SD-ASD energetics, preferred spacer lengths, and local folding windows).

### Future Work

For fine-grained control of translation initiation, we will extend this approach by codesigning or empirically calibrating neighboring UTR elements and coding-sequence features alongside the SD cores from the activity clouds, using the activity cloud selection as a starting point.

Specifically, we plan to determine the impact of the spacer length, local mRNA secondary structure, and nucleotide composition immediately upstream and downstream of the SD region to quantify their combined effects on ETR.

Further work will involve performing thermodynamic calculations, which we expect to show that many seemingly unrelated cores reach indistinguishable hybridization energies. In silico folding analysis could further indicate that cloud members adopt comparable hairpin topologies that leave the core similarly exposed, providing a structural route to this functional degeneracy.

These results will feed a machine learning model trained to predict ETR from composite sequence features, allowing the in silico screening of candidate RBS-UTR-coding combinations prior to experimental validation. Finally, we will benchmark the extended framework across diverse gene contexts and host strains to evaluate its generalizability and refine context-dependent design rules for precision tuning of protein expression in synthetic biology applications.

## Methods

We targeted the Shine–Dalgarno core sequence within the ribosome-binding site, owing to its role in translation initiation in *E. coli*. It is one of the determinants that governs the rate-limiting step of translation initiation through hybridization with the 16S rRNA ASD sequence, which helps adjust the initiation codon to the ribosomal P-site.^1^

### Data Sets

We utilized ETR measurements from the EMOPEC.^3^ The EMOPEC data set biologically represents ETR for a comprehensive set of SD core sequences. These values quantify the contribution of specific 6-base core SD sequences to protein expression levels, with variations attributed solely to differences in translational efficiency. The experimental context involved comprehensively mutating the six-base SD sequence of a constitutive promoter driving a GFP gene integrated into the *E. coli* K12 MG1655 genome. All variants of these SD sequences were assayed in an identical genetic context—fixed 5′-UTR architecture (RBS length), spacer sequence and length, start codon, and reporter coding sequence, so that only the SD motif varied. Measurements were obtained via Flow-seq, combining fluorescence-activated cell sorting with next-generation sequencing, performed in controlled conditions.^3^ This data set serves as an empirical map of translation efficiency.

The data set is available for download in the Supporting Information section as a part of the EMOPEC software (https://github.com/micked/EMOPEC).

The data are provided as two Python scripts, each containing dictionaries that map core sequences to their corresponding ETRs. Given that the sequence context was held constant across all experiments, variations in measured RBS strengths can be attributed primarily to differences in translational efficiency arising from changes in the SD core sequences; thus, we treat RBS strength as a proxy for ETR, while acknowledging that inherent biological and measurement noise may introduce residual variability.

The first script contains data from the experimental measurements, and the second one contains predicted data from the authors for core sequences where the measurements were not confident enough (these predicted data constitute 25% of the data set). This prediction was made using a Random Forest regressor model, where the 5-fold cross-validation of this model yielded an *R* ^2^ of 0.89. An out-of-bag score also showed an *R* ^2^ of 0.90, indicating a strong fit of the model to the data. We combined these two data sets into a single one, using the real measurement if available and the predicted one otherwise.

The integral-flipping metric (IFP_0–480 min_) of the first replicate for the whole 300k large data set was used from the μASPIre data set.^11^ It can be downloaded from https://github.com/JeschekLab/uASPIre. The integral-flipping metric represents the normalized integral of the flipping profile between 0 and 480 min after induction, serving as a quantitative descriptor of RBS activity that correlates with cellular protein levels. This metric was used since the authors observed a high correlation with the GFP fluorescence and devised this metric specifically for comparison.

### Tree Construction and Pruning

Using only the EMOPEC data set, we created a Python Jupyter notebook that constructed the hierarchical tree and performed the pruning.

We build a tree over all 4096 six-nucleotide SD cores starting from a root placeholder “_____” (level 0, no fixed nucleotides). Contrary to a left-to-right scheme that would yield only four children at level 1, our hierarchy fixes one additional position at any index in the 6-mer at each level. Thus, a level *k* node is any length-6 pattern with *k* fixed nucleotides and (6–*k*) underscores; from such a node, we choose any underscore and assign one of {A, C, G, T}.

Each level *k* node therefore has (6–*k*) × 4 children, and the total number of nodes at level *k* is (6k)⁡4k . Specifically, level 1 has 6 × 4 = 24 nodes, e.g., A_____, _A____, ..., _____G, _____T. Level 2 includes all patterns with two fixed positions (e.g., AC____, A_G___, ...). This process continues until level 6, which contains all fully specified cores (no underscores).

Each sequence (leaf or internal node) was represented by an RBSSequence object (defined in common.py) that stored:

1. The nucleotide pattern (string of length 6, mixing fixed bases and underscores).
2. The mean ETR value associated with that pattern (sourced from the EMOPEC data set), together with the CV of the child ETRs.
3. Pointers to its child sequences (empty for leaves).

Once all 4096 leaf sequences (fully specified 6-mers) and all the intermediate (partially specified) sequences were instantiated, we computed the mean and CV of their descendant ETRs at each internal node.

Then a pruning was performed iteratively, level by level (underscores_count = 1 to 6, starting from level 5 with a single underscore and going up). For each number of underscores *k* (starting with *k* = 1):

1. We collected all nodes containing exactly *k* underscores (i.e., all incomplete patterns of length 6 with *k* variable positions).
2. For each such node, we counted how many child sequences (one level down) it possessed. These counts were compared to a predefined maximum allowed child count, MAX_COUNTS­[*k*], which is the initial number of occurrences in the unpruned tree, i.e., the exact original number of children at depth *k* in the fully expanded tree. This threshold is dictated by the unpruned tree’s inherent branching structure: sequences containing a single underscore occur exactly once, whereas those with two underscores occur twice (for instance, “AA____” can be produced both via “A_____” and via “_A____”).
3. If a node’s number of children exceeded MAX_COUNTS­[*k*], its children were ranked by their individual CV contributions (via a Counter on the node.children list). Starting from the child with the highest CV contribution, we removed children one at a time—updating the parent’s CV after each removal. Removal was implemented by deleting the child object from both the parent’s children list and the master all_sequences list. The remaining sequences were resorted by CV at the end of each level.

We selected the CV as a metric for pruning since CV normalizes variability by the mean. Therefore, it provides a scale-invariant measure that highlights relative fluctuations, especially when mean ETR levels differ or exhibit heteroscedasticity. Thereby, it reflects proportional dispersion more accurately than absolute variance or simple mean-difference metrics.

4. The pruned children were not expanded in subsequent rounds; only surviving children were eligible for further pruning at deeper levels.

After completing the pruning for all six levels, the resulting tree contained a reduced set of internal nodes, each representing a subspace of SD cores whose descendants exhibited a relatively narrow ETR distribution. We then generated a CSV file listing every root-to-node path alongside its corresponding relative strength (mean ETR) by traversing remaining children from the root. Each CSV row had seven columns for levels 0–6 (i.e., the six sequential nucleotide positions plus the final node), followed by the relative strength of that node, which was looked up in EMOPEC_DATA. These files (CV dictionary and path CSV) were used directly for downstream DBSCAN clustering and experimental validation steps described in the next section.

The complete tree construction and pruning workflow is archived in 10_tree_creation_and_prunning.ipynb, and resultant summary files directly informed the downstream clustering algorithm.

### Activity Cloud Identification

To define discrete “activity clouds” of SD-core variants with similar relative expression levels, we applied density-based spatial clustering (DBSCAN) to the data set containing pruned branches obtained from the hierarchical tree.

DBSCAN was chosen because it can discover clusters of arbitrary shape and explicitly label sparse points as noise, preventing outliers from distorting true “activity clouds” in feature space. It requires only two intuitive parameters (ϵ and min_samples) rather than a fixed cluster count, and its density-based grouping aligns naturally with iso-energetic basins in the SD-ASD free-energy landscape, yielding interpretable, robust clusters for downstream analysis. In contrast, hierarchical methods can inadvertently split or merge these basins if branch distances do not map cleanly onto energetic neighborhoods.

1. Data loading and initial filtering. We began by reading the CSV data file, which contains one row for every node retained after pruning. Each row includes seven columns for the root-to-node nucleotide path (levels 0–6), a column SEQ (the fully specified six nt sequence at that node), and a column CORE_REL_EXPR (the mean ETR for that sequence, drawn from the EMOPEC data set). For clustering purposes, we discarded column level 0 (since the root sequence is always the same).
2. Feature encoding (one-hot plus expression). Each six-nucleotide sequence (*s*_1_ *s*_2_, ..., *s*_6_) was represented by a 30-dimensional one-hot vector. In this encoding, each nucleotide position *i* (for *i* = 1, ..., 6) contributes five binary variables corresponding to {A, C, G, T, _}. Concretely, for a given sequence *s*_1_ *s*_2_, ..., *s*_6_, we construct a binary vector **x** ∈ {0,1}^30^ such that, within the block of five entries associated with position *i*, exactly one entry is set to 1—namely, the entry corresponding to the nucleotide *s*_*i*_—and all other entries in that block are 0. Here, the symbol “_” denotes a wildcard, meaning that any nucleotide could occupy that position.

In addition to the nucleotide encoding, the variable CORE_REL_EXPR, a floating-point ETR value in [0,1], was transformed using cumulative binary encoding into a 200-dimensional vector **y** ∈ {0,1}^200^. First, this value *v* is rounded to two decimal places. Then the number of ones, *k*, is computed as

k=⌊100·v+0.5⌋=round(v,2)×200

and **y** is formed by placing *k* ones in the first *k* entries and zeros in the remaining 200 – *k* entries. In other words, if *v* = 0.50, then *k* = 0.50 × 200 = 100, and

$$y=\left[\underset{100}{\underset{︸}{1,1,...,1}},\underset{100}{\underset{︸}{0,0,...,0}}\right]$$

This cumulative scheme ensures that larger expression values correspond to longer runs of ones, thereby normalizing CORE_REL_EXPR so its scale is comparable to the nucleotide-based features during clustering, balancing the numeric “weight” of the relative expression against the one-hot sequence features. It also preserves the natural ordering of expression values as exact Hamming distances, and it yields more interpretable, better separated clusters than a lone normalized float ever could.

No other numeric features required discretization.

- 3.  Parameter sweep for DBSCAN. We employed scikit-learn’s DBSCAN to cluster the data. The distance metric was Euclidean over all dimensions. To determine an appropriate neighborhood radius (eps), we performed a sequential sweep:
- Initialize eps at 5.9.
- Run DBSCAN with min_samples = 1 (permitting singleton clusters) and default leaf_size = 30.
- Record the number of distinct labels (clusters plus noise, where noise is labeled −1).
- Increment eps by 0.05 and repeat, until the total number of labels is ≤400.

The 400-cluster cap was arbitrarily chosen to balance granularity and statistical robustness. It is large enough to resolve individual activity clouds for fine selection, yet small enough that each cluster retains a sufficient number of sequences for reliable estimates and easy interpretation. Singleton cluster formation was enabled to enhance the homogeneity of the activity clouds by segregating outliers into their own clusters. As a result, 55 activity clouds each comprised a single SD core sequence.

At each step, clustering was invoked with only the final eps (no additional parameter changes), and the loop terminated when the cluster count threshold was satisfied. The min_samples = 1 choice ensured that even a single outlier could form its own cluster rather than being labeled as noise. The final eps value was retained for the definitive clustering assignment.

4. Cluster label assignment and noise handling. After convergence of the eps sweep, each node received an integer cluster label (0, 1, 2, ...).
5. Postclustering aggregation. We aggregated cluster membership and expression statistics as follows:
(a) For each cluster *C*_*j*_, collect the set of six-nt sequences $\left\{s_{1}^{\left(j\right)},s_{2}^{\left(j\right)},...\right\}$ belonging to *C*_*j*_.
(b) Compute the mean of CORE REL EXPR over all nodes in *C*_*j*_, denoted μ_*j*_, and the standard deviation σ_*j*_.
(c) Concatenate the sequences in *C*_*j*_ into a single comma-separated string.

The results were written to data/processed/clusters_with_stats.csv, which contains one row per cluster with the following columns:

CLUSTER: the integer label *j*.
CORE REL EXPR_mean: μ_*j*_.
CORE REL EXPR_std: σ_*j*_.
SEQ_unique: comma-separated list of all six-nt sequences in cluster *C*_*j*_.
6 .Optional visualization. To verify that clusters correspond to coherent “bands” (i.e., clouds) of relative expression, we generated a two-dimensional scatter plot of each node’s index (row number in the pruned list) versus its CORE REL EXPR, coloring each point by its cluster label (with noise points plotted in red using a distinct marker). The plot (Figure 10) confirmed that clusters align as horizontal or slightly diagonal bands along the expression axis, indicating that DBSCAN successfully grouped nodes with similar relative translation rates.

**Figure 10:**
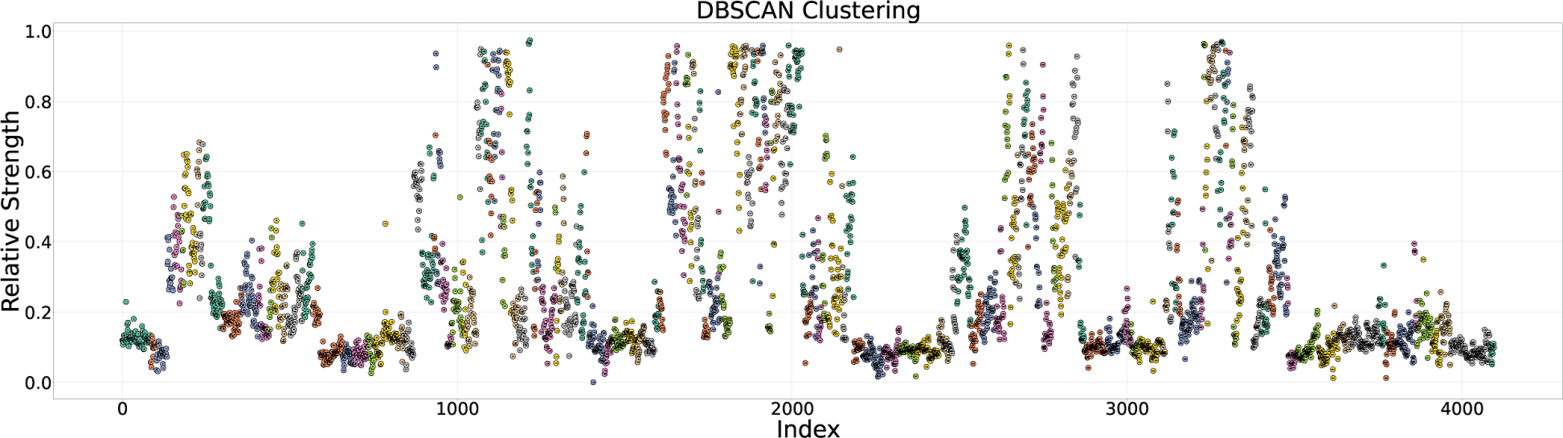
High-level visualization of all 4096 six-mer SD sequences and their relative ETRs. Nodes are ordered by their position in the hierarchical tree using a depth-first traversal. Each circle corresponds to one SD sequence; its color (and the numeral inside) indicates the assigned activity cloud. Regions of adjacent branches with consistently high or low ETRs highlight early positional effects that emerge at level 1–2 of the hierarchy (i.e., after fixing one or two positions), consistent with strong nucleotide-by-position influences on initiation in *E. coli*.

The complete DBSCAN clustering workflow is archived in 20_DBSCAN_clustering.ipynb, and resultant summary files directly informed downstream activity cloud selection for experimental validation.

Each activity cloud created in this step represents a set of SD core sequence variants expected to yield equivalent translation rates, effectively grouping together sequences that fall within the same iso-energetic basin of translation efficiency.

### Web Interface Implementation

To facilitate interactive exploration of the SD core sequence space, we implemented a lightweight web interface that integrates the pruned hierarchical tree and activity cloud assignments. The application was developed using Flask in Python and dynamically loads the CSV outputs generated from the tree-pruning and DBSCAN clustering workflows.

The interface allows users to

- Navigate through the pruned hierarchical tree, expanding and collapsing nodes to inspect partially specified motifs and their descendant sequences.
- Visualize activity clouds as interactive scatter plots with color-coded cluster memberships and tooltips displaying each sequence’s mean ETR and CV.
- Generate and download cluster-specific summaries, including the sequence composition and expression statistics for each activity cloud.

The frontend relies on standard web technologies (HTML5, JavaScript, and Plotly based visualizations) to provide an intuitive experience. All data required for rendering is precomputed and stored in compact CSV format (loaded into memory when the app starts) to ensure rapid loading without additional server-side processing.

The web interface can be accessed at http://sb2cl-vm1.ai2.upv.es/ for public use. The full source code and deployment instructions are archived in the same GitHub repository as the analysis notebooks, ensuring complete reproducibility of the results presented in this work.

### Validation

To assess how well our predictions based on the activity clouds correspond to measurements from μASPIre,^11^ we identified the RBS core sequence in each RBS sequence provided in the data set, determined the cluster in which this core sequence is, and compared it with the cluster mean ETR.

1. Data acquisition and parsing. The raw μASPIre data set was used, as described in the Data Sets section.

Upon loading this file into a pandas DataFrame (uaspire_df), we filtered out any rows with Read_Count < 500 to ensure reliable measurements, yielding a cleaned set of SD-core variants.

2. Matching activity clouds predictions to μASPIre. A simple algorithm was implemented to identify the RBS core within each RBS sequence in the μASPIre data set. For spacer sequences 5–8 nt long, we slide a six-nucleotide window across the upstream region, calculate the ETR for each possible segment, and then designate the segment with the highest ETR as the RBS core.

Next, we loaded the assembled data set containing activity-cloud information, with one record per cloud. Each RBS sequence in the μASPIre data set was then annotated with its predicted ETR based on the activity cloud associated with its identified RBS core.

3. Correlation analysis. We assessed the linear association between the IFP_0–480 min_ and the ETR of each determined activity cloud by computing the Pearson correlation coefficient, using the pearsonr method from Python’s stats package.
4. Error-band assessment. Since we obtained multiple records in the μASPIre data sets belonging to the same activity clouds, we also computed the mean values of IFP_0–480 min_ together with the 95% confidence interval. The mean and standard deviation was computed via data aggregation of the pandas data set and the 95% CI was computed from the standard deviation as 1.96*yerr/np.sqrt­(count), where yerr is the standard deviation and count is the number of records in the same activity cloud.

### Validation against the RBS Calculator (Data Set Construction and Quantile-Based Sampling)

We validated our coarse-grained predictions by comparison with the RBS Calculator.^9^ As a template, we used the experimentally characterized 5′-UTR from our plasmid pIAKA1_11 that constitutively expresses GFP, with a fixed 7 nt SD-AUG spacer and ATG start codon. From this template we generated the full combinatorial set of variants obtained by substituting all six nucleotides immediately upstream of the spacer (4096 six-mers). For each variant, we identified the SD core as the 6-mer within the canonical search window that yielded the most favorable SD-ASD hybridization free energy (Δ*G*_SD‑aSD_), permitting SD-AUG spacers of 5–8 nt; the corresponding cloud mean ETR for that core was then retrieved from our activity-cloud map.

To stratify variants by local mRNA structure, we computed a standardized measure of folding stability for the RBS region (fixed window centered on the SD/spacer; same window for all variants). The resulting distribution of folding free energies (Δ*G*_fold_) was used to select the representative sequences. Weak-structure variants were taken from the variant with the least negative Δ*G*_fold_, and strong-structure variants from the sequences with the most negative Δ*G*_fold_. From each subset, we sampled *n* = 5 representative sequences for downstream comparison, based on the quantiles of their mean ETR (to cover its full range). For each sampled sequence, we queried the RBS Calculator to obtain its predicted translation initiation rate and plotted this value against the cloud-mean ETR of the identified SD core (Figure 6). All enumeration, SD core identification (including the 5–8 nt spacer constraint), quantile selection, and plotting are implemented in the accompanying Jupyter notebook (80_rbs_calc_validation.ipynb), enabling reproduction or reanalysis with alternative quantile thresholds or window definitions.

### Mutational Graph Construction, Metrics, and Visualizations

#### Data and Preprocessing

We used the table of SD variants with columns SEQ_unique (the SD core sequence), CORE REL EXPR_mean (ETR), and CLUSTER_ (activity-cloud label). Columns were renamed to core, etr, and cloud, and validated.

#### Sequence Graph

We constructed an undirected graph whose nodes are observed cores, with edges that connect pairs at Hamming distance 1 (single-nucleotide difference, with a maximum of 18 neighbors per node). Each node stores its etr and cloud. For intracloud analyses, we considered the subgraph induced by all cores in a given cloud.

#### Intracloud Robustness

For each cloud-induced subgraph we computed the number of nodes/edges, number of connected components, giant-component size and giant-component fraction (giant/total), average degree, average clustering coefficient, and average shortest-path length on the giant component.

#### Intercloud Meta-Graph and Boundary Steepness

We formed a meta-graph over clouds with an undirected edge if any one-step bridge connects cores across the two clouds. For each meta-edge, we recorded the bridge count and the mean absolute |ΔETR| across bridges with finite ETR at both ends. These quantities were assembled into cloud × cloud matrices for visualization.

#### Evolutionary Accessibility

From all undirected sequence-graph edges we counted directed neighbor moves between cloud pairs and built a row-normalized transition matrix *P*(*C*_*i*_ → *C*_*j*_) (diagonal gives *P*(stay); 1-diagonal gives *P*(leave)). In parallel, we computed, for each ordered pair, the beneficial-move fraction (proportion with ΔETR > 0). For selectively accessible paths, we constructed a directed graph that retains only edges with ΔETR ≥ 0 (also tested strict >0) and reported reachability and shortest path lengths between specified source/target clouds.

#### Visualization

The visualization was achieved by heatmaps: (i) bridge counts, (ii) mean |ΔETR|, (iii) *P*(*C*_*i*_ → *C*_*j*_), and (iv) beneficial fractions. Where helpful, rows/columns were ordered by mean cloud ETR. Plots were generated with Matplotlib/Seaborn; graph computations used NetworkX. All analysis code is provided in the accompanying Jupyter notebook 70_sequence_variations.ipynb.

#### Data Analysis

All statistical analyses, tree building, pruning, and clustering were performed in Python 3.13.1 with pandas 2.3.1, using SciPy 1.16.0 for hierarchical clustering, NumPy 2.3.1 for numerical operations, and scikit-learn 1.7.0 for DBSCAN implementation. The Jupyter notebooks used are available for download at https://github.com/sb2cl/Activity-Clouds.

## Supplementary Material



## Data Availability

The SI PDF document is provided with this submission and mirrored in the project repository (see Data/Code Availability). All data underlying this study are available at https://github.com/sb2cl/Activity-Clouds.

## Notes

Accompanies this article and provides extended figures and reproducible code. It is intended to facilitate verification of results and enable readers to build on our analyses (PDF)

## Abbreviations

ASD: anti-Shine–Dalgarno sequence
CI: confidence interval
DBSCAN: density-based spatial clustering of applications with noise
DBTL: design-build-test-learn
ETR: effective translation rate
GFP: green fluorescent protein
IFP: integral flipping profile
mRNA: messenger ribonucleic acid
rRNA: ribosomal ribonucleic acid
RBS: ribosome binding site
SD: Shine–Dalgarno sequence
UTR: untranslated region
