# Supplementary material for: Activity-Cloud Organization of Shine–Dalgarno Sequences to Guide Translation Engineering in Escherichia coli
Source: ACS Omega. 2026 Feb 1;11(6):9607–18. doi: 10.1021/acsomega.5c09784 (PMC12917692; doi:10.1021/acsomega.5c09784)
Supplement: Supplementary file 1 [file ao5c09784_si_001.pdf]

Supplementary Information

for

Activity-Cloud Organization of Shine-Dalgarno Sequences to Guide Translation Engineering in E. *coli*

Pavel Zach[1], Yadira Boada[1], Jesús Pico[1], Alejandro Vignoni[1,*]

[1]Synthetic Biology and Biosystems Control Lab, Instituto de Automática e Informática Industrial, Universitat Politècnica de València, València, Spain.

∗ Correspondence: <vignoni@isa.upv.es>

This PDF file includes:

- Supplementary Figures S1–S3, with captions
- Description of the Web Application
- Data and Code Availability

Contents

S1 Supplementary Figures **2**

S2 Description of the Web Application **4**

S2.1 Browse Sequences 4

S2.2 Sequence → ETR 4

S2.3 ETR → Sequence 4

S2.4 Browse Clouds 5

S2.5 Tree View 5

S2.6 Clouds View 5

S3 Data and Code Availability **5**

S1 Supplementary Figures

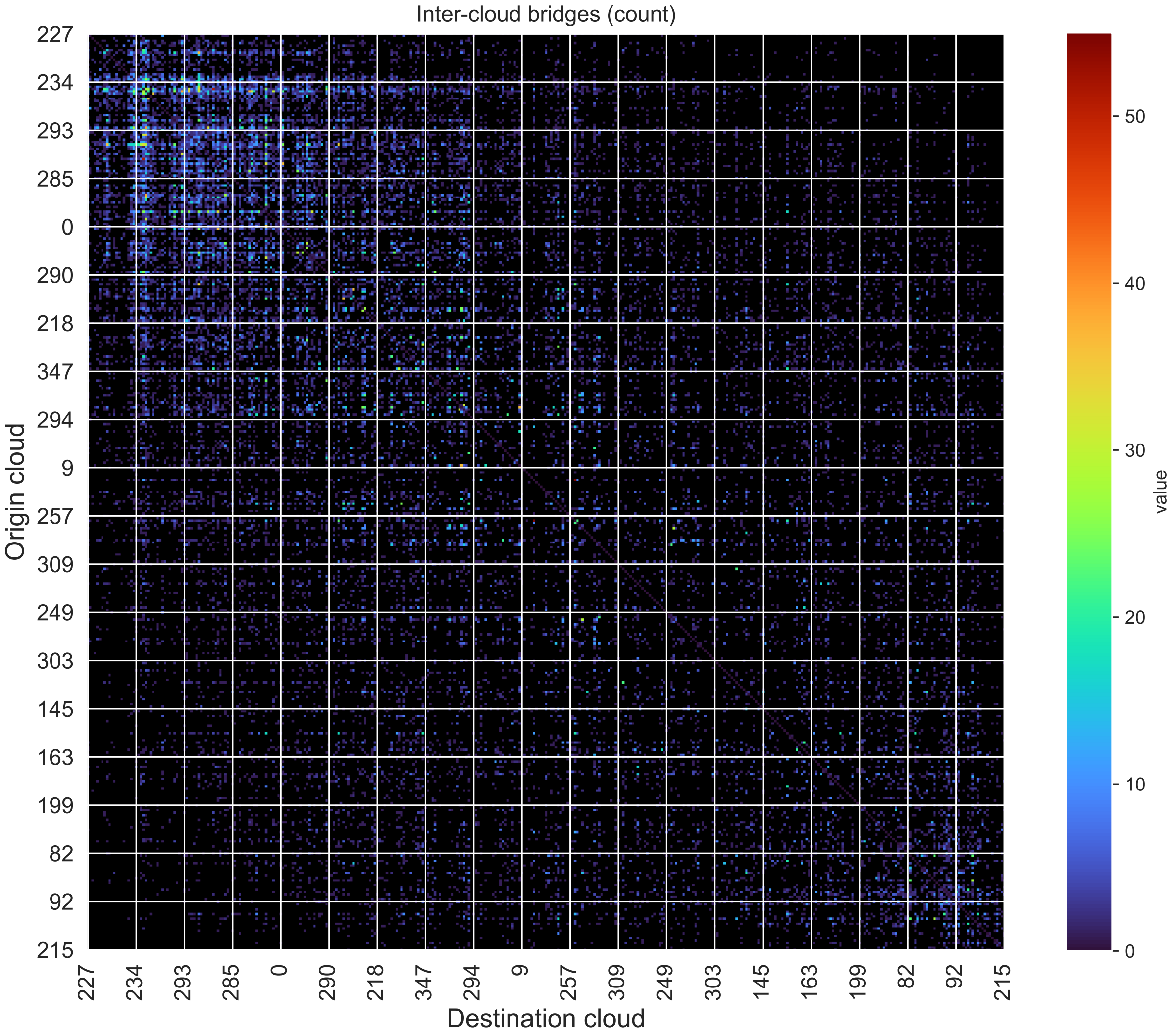

Figure S1

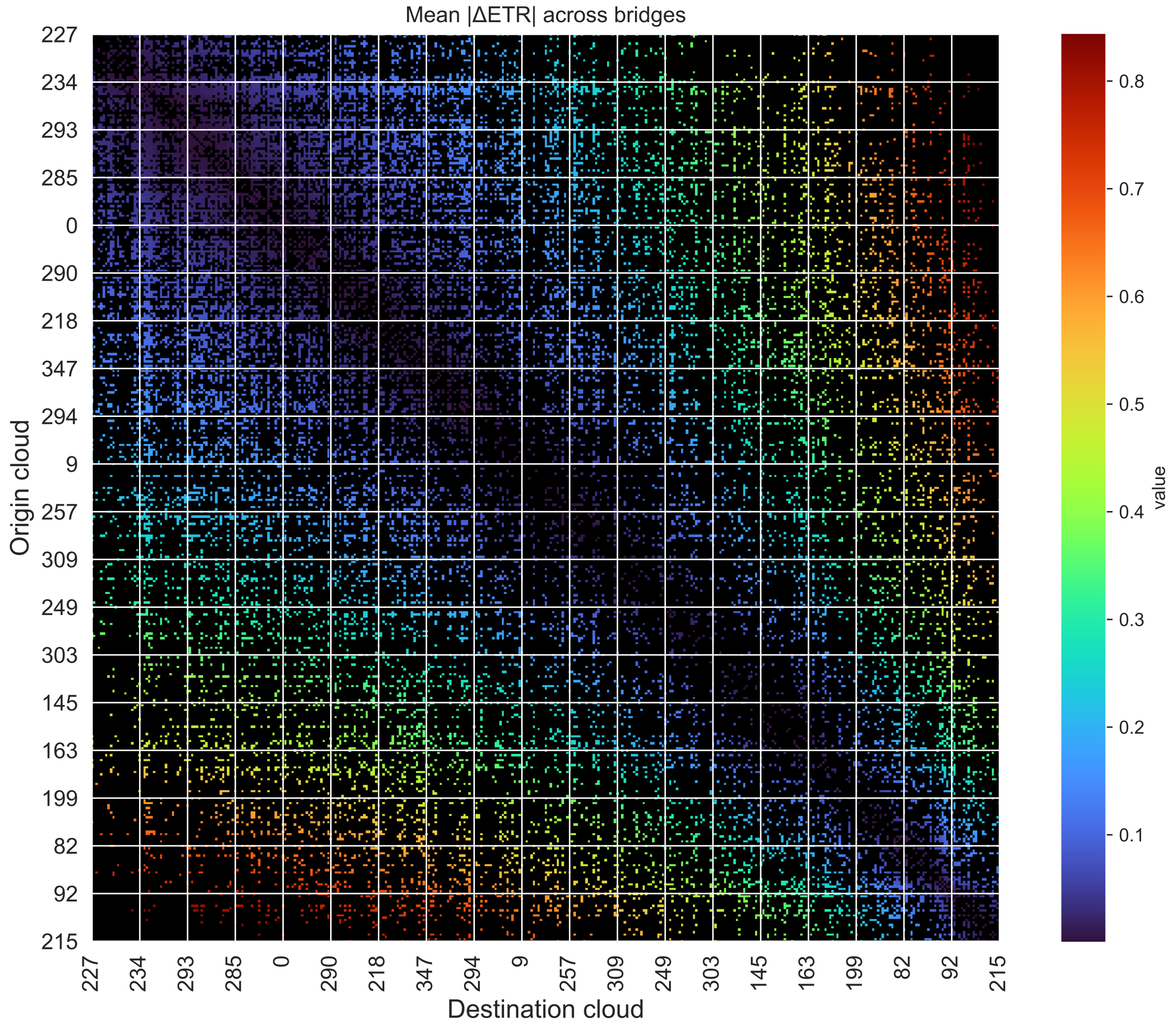

Mean |ΔETR| across bridges
Origin cloud
227
234
293
285
0
290
218
347
294
9
257
309
249
303
145
163
199
82
92
215
Destination cloud
value
0.1
0.2
0.3
0.4
0.5
0.6
0.7
0.8

Figure S2

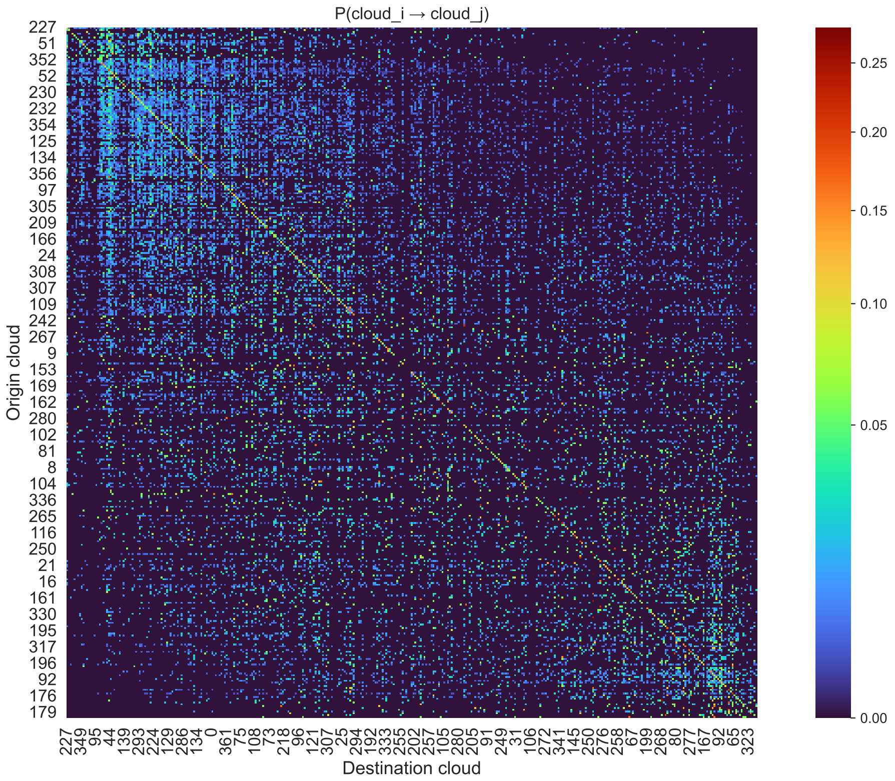

Figure S3

S2 Description of the Web Application

S2.1 Browse Sequences

This page reports summary statistics for all SD 6-mer sequences that match the current pattern. By default, it considers the full set of 4,096 sequences. Users can restrict the set by replacing underscore wildcards ("_") with specific nucleotides. The histogram and summary statistics update instantly as the pattern is refined.

S2.2 Sequence → ETR

Given a full 5'-UTR input (without the start codon), the app locates the SD core within the canonical window and decomposes the sequence into upstream region, SD core, and SD–AUG spacer. The identified core is then mapped to its *activity cloud*, displaying the cloud's mean ETR and its confidence interval.

S2.3 ETR → Sequence

Given a target ETR in $[0, 1]$, the app returns the best-matching *activity clouds* and representative SD cores from those clouds. Results include each cloud's mean ETR and example cores.

S2.4 Browse Clouds

This table lists all SD cores with their assigned activity cloud, activity cloud statistics , and the hierarchical "path" from the root placeholder to the fully specified 6-mer.

S2.5 Tree View

This view visualizes the pruned hierarchy from Level 0 (center) down to fully specified SD cores. Clicking a node expands one level deeper along that branch; double-clicking collapses to its ancestors. Tooltips summarize node content (fixed positions, examples) and link to the corresponding cloud/sequence details.

S2.6 Clouds View

This interactive plot summarizes activity clouds and their mean relative expression. Hovering over a cloud reveals its statistics (mean ETR, confidence interval, size).

S3 Data and Code Availability

Raw and processed data, together with the code used to generate the results in this SI, are available at: `https://github.com/sb2cl/Activity-Clouds`. Access details, licenses, and any restrictions are described there.
